# Tryptophan regulates the expression of IGFBP1 in bovine endometrial epithelial cells in vitro via the TDO2-AHR pathway

**DOI:** 10.1186/s12917-024-04191-9

**Published:** 2024-09-04

**Authors:** Peng-Chao Wang, Ze-Kun Liu, Jia-Rong Li, Zi-Hui Zhao, Qian-Wen Chang, Xiao-Min Guo, Lin Jin, Yong-Ting Hu, Zhenshan Yang

**Affiliations:** 1 College of Veterinary Medicine, Shanxi Agricultural University, Taigu, 030801 China; 2 College of Veterinary Medicine, South China Agricultural University, 483 WuShan Road, Guangzhou, 510642 Guangdong China

**Keywords:** Bovine, Kynurenine, PGE2, Tryptophan, Uterine receptivity

## Abstract

**Background:**

This study aimed to identify the roles of L-tryptophan (Trp) and its rate-limiting enzymes on the receptivity of bovine endometrial epithelial cells. Real-time PCR was conducted to analyze the differential expression of genes between different groups of bovine endometrial epithelial cells. Western blot was performed to detect Cyclooxygenase-2 (COX2) expression after treatment with Trp or kynurenine (the main metabolites of Trp). The kynurenine assay was used to examine if Trp or prostaglandin E2 (PGE2) can increase the production of kynurenine in the bovine endometrial epithelial cells.

**Results:**

Trp significantly stimulates insulin growth factor binding protein 1 (IGFBP1) expression, a common endometrial marker of conceptus elongation and uterus receptivity for ruminants. When bovine endometrial epithelial cells are treated with Trp, tryptophan hydroxylase-1 remains unchanged, but tryptophan 2,3-dioxygenase 2 (TDO2) is significantly increased, suggesting tryptophan is mainly metabolized through the kynurenine pathway. Kynurenine significantly stimulates *IGFBP1* expression. Furthermore, Trp and kynurenine significantly increase the expression of aryl hydrocarbon receptor (*AHR*). CH223191, an AHR inhibitor, abrogates the induction of Trp and kynurenine on *IGFBP1*. PGE2 significantly induces the expression of *TDO2*, *AHR*, and *IGFBP1*.

**Conclusions:**

The regulation between Trp / kynurenine and PGE2 may be crucial for the receptivity of the bovine uterus.

**Supplementary Information:**

The online version contains supplementary material available at https://doi.org/10.1186/s12917-024-04191-9.

## Background

The success rate of fertilization in ruminants after mating or artificial insemination is high. However, the birth rate was significantly lower due to embryo loss and embryo implantation failure in early pregnancy [1]. Conceptus implantation is a process of conceptus-endometrium crosstalk during early pregnancy [2–5]. Receptive endometrium is essential for conceptus implantation and successful pregnancy [4]. Scientists explored some genes as potential early pregnancy diagnostic markers in the endometrium. Insulin growth factor binding proteins (IGFBPs) are likely to play an essential role in regulating a uterine environment that promotes the growth and development of the conceptus to implantation [6, 7]. IGFBP1, one of the mammalian IGFBPs family, is a standard endometrial marker of uterine receptivity and conceptus elongation for ruminants [8]. PGE2 plays a pivotal role in endometrial receptivity [9]. Furthermore, PGE2 participated in the growth and elongation of the ruminant conceptus [10]. However, there is poorly defined information underlying the molecular mechanism of PGE2 in regulating uterine receptivity and endometrial epithelial cell markers in ruminants.

Adequate maternal nutrition is vital for embryonic development and maternal fertility [11–13]. As nutrients, various amino acids are taken from the maternal uterus to promote conceptus growth during bovine early pregnancy [14]. Tryptophan (Trp), as a nutrient and essential amino acid, is involved in protein synthesis and many critical physiological functions, including decidualization, embryo immune tolerance, and fetal growth [15–17]. Only 5% Trp is formed to serotonin (5-HT) by tryptophan hydroxylase 1 (TPH1) in the 5-hydroxytryptamine pathway during tryptophan metabolism [16]. 95% tryptophan is metabolized to kynurenine through the rate-limiting enzyme of indoleamine 2,3-dioxygenase (IDO) and tryptophan 2,3-dioxygenase (TDO) in the kynurenine pathway [17]. As a ligand, kynurenine can bind and activate aryl hydrocarbon receptors (AHR) and induce tolerance [16, 18]. However, the regulation and function of tryptophan and kynurenine for uterus receptivity are still poorly understood. AHR is regulated by progesterone and estradiol in the uterus [19]. Moreover, AHR regulates the embryo’s nourishment and maintains pregnancy [20]. The mechanisms by which the AHR regulates bovine uterus receptivity are poorly unknown.

This study examined the effects of tryptophan and kynurenine on bovine endometrial epithelial cells. Our data indicated that tryptophan and kynurenine promote *IGFBP1* expression to enhance endometrial receptivity via PGE2-TDO2-AHR signaling.

## Results

### IGFBP1 expression in the bovine endometrial tissue

**Fig. 1:**
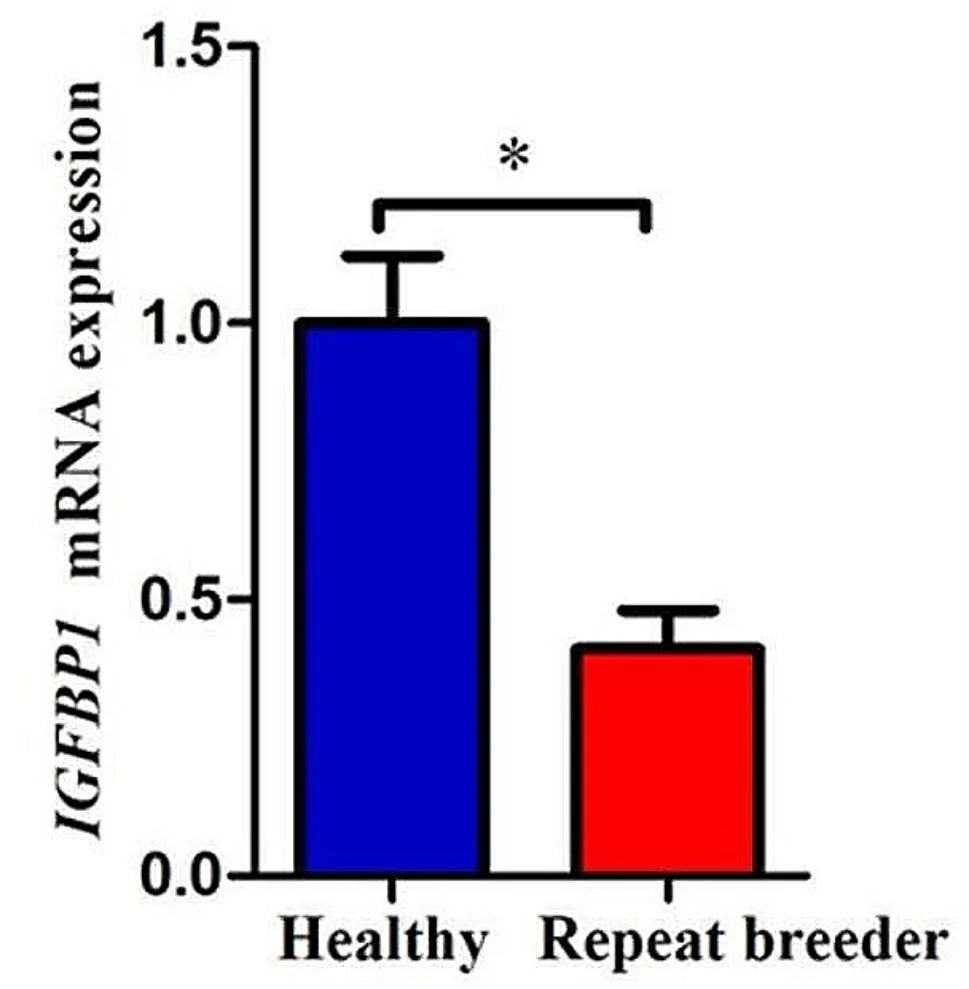
Comparison of *IGFBP1* mRNA isolated from repeat breeder and healthy bovine in uterus endometrium. *P < 0.05. Data were normalized with *GAPDH*

We first used real-time PCR to detect *IGFBP1* mRNA in the bovine uteri of repeat breeder and normal. As a marker of potential ruminant endometrial [8], *IGFBP1* mRNA abundances were remarkably greater for normal cows than repeat breeder cows in endometrial (Fig. 1).

### Effect of Tryptophan treatment on the expression of IGFBP1 in bEECs

**Fig. 2:**
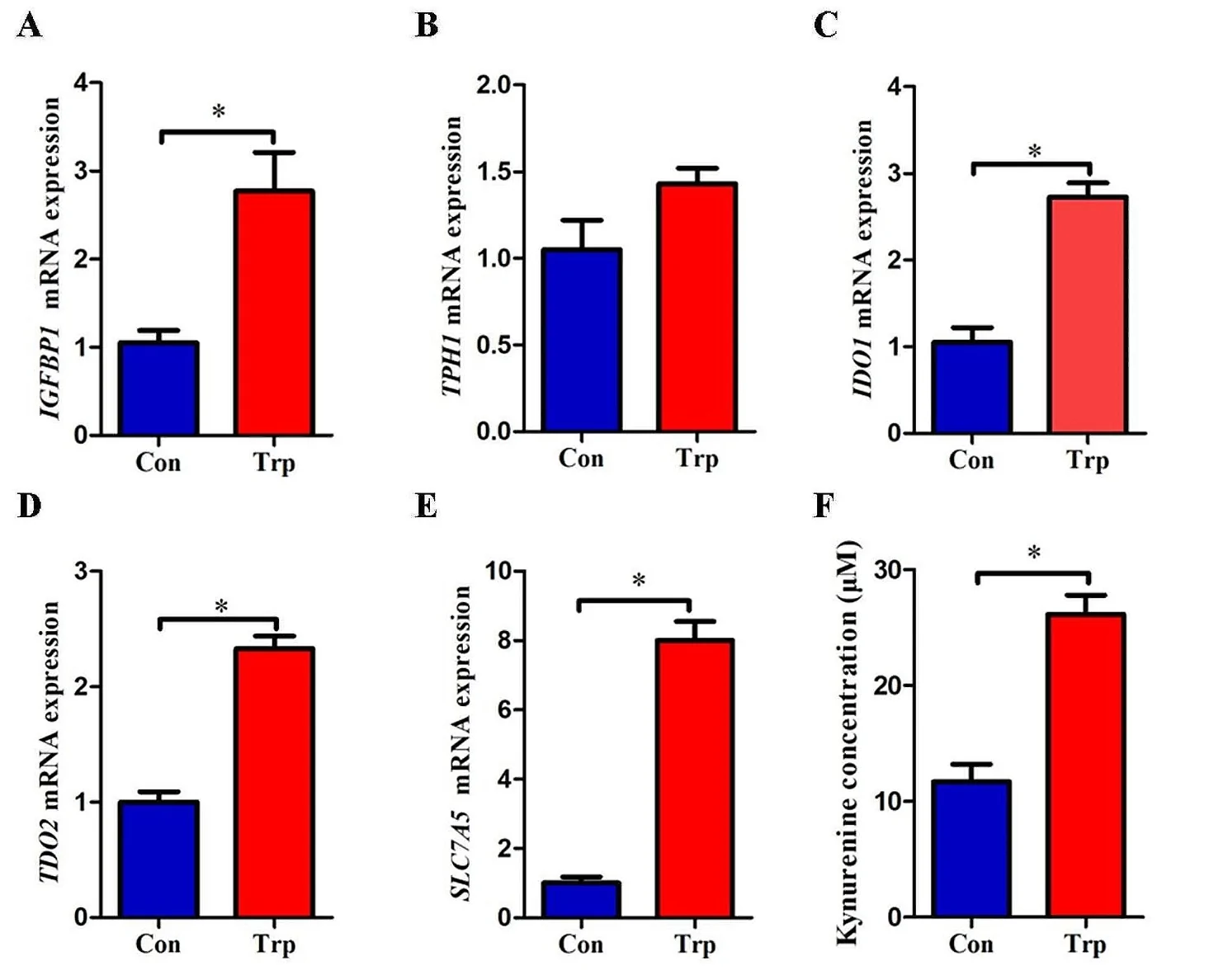
Effects of Tryptophan in bEECs. (**A**) The level of *IGFBP1* mRNA. (**B**) The level of *TPH1* mRNA. (**C**) The level of *IDO1* mRNA. (**D**) The level of *TDO2* mRNA. (**E**) The level of *SLC7A5* mRNA. (**F**) Kynurenine concentration. *P < 0.05. Every experiment was repeated at least thrice independently. Con, control; Trp, tryptophan

To investigate whether tryptophan affects the gene that regulates endometrial receptivity processes in cattle, we treated bEECs with tryptophan. We found that *IGFBP1*, a reliable marker for bovine in uterine receptivity [8], was remarkably upregulated by tryptophan (Fig. 2A). To explore the tryptophan metabolism, we examined *TPH1* (a key enzyme for the serotonin pathway) and *IDO1* /*TDO2 *(key rate-limiting enzymes for the kynurenine pathway) in tryptophan-treated cells. After epithelial cells were treated with tryptophan, *TPH1* mRNA remained unchanged (Fig. 2B). The mRNA levels of *IDO1* and *TDO2* were significantly increased (Fig. 2C and D), suggesting that tryptophan may be mainly metabolized to kynurenine. As an amino acid transporter, solute carrier family 7 member 5 (SLC7A5) could transport tryptophan from the extracellular to the intracellular of the cell [21]. In the cattle, expression of *SLC7A5* was increased in the endometrium as the estrous cycle [22]. So, we wonder whether tryptophan affects the expression of SLC7A5. Our results suggested that *SLC7A5* expression was significantly stimulated by tryptophan (Fig. 2E). Kynurenine concentration was also increased in the tryptophan treatment group (Fig. 2F). These data may suggest that tryptophan may be transported to the intracellular by SLC7A5 and metabolized through the kynurenine pathway in bEECs.

### Effect of kynurenine on the endometrial receptivity in bEECs

**Fig. 3:**
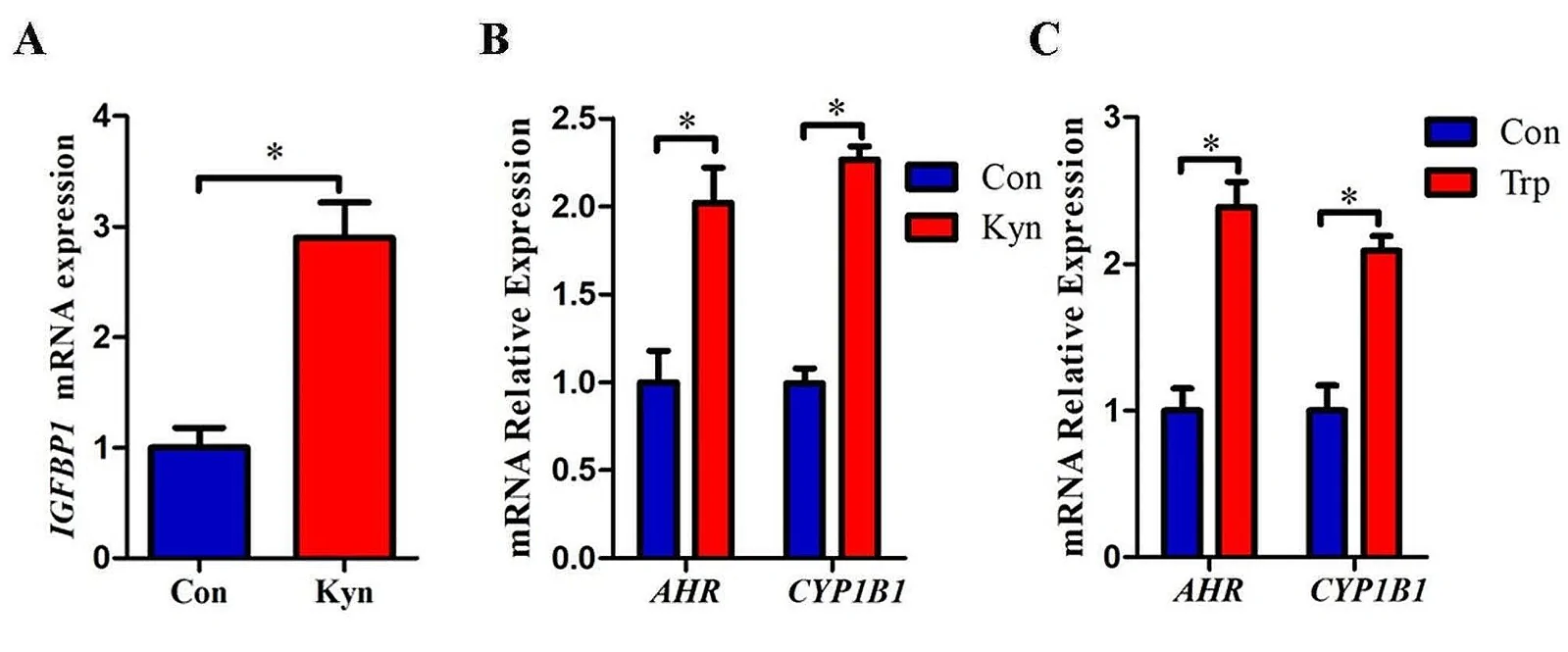
Effects of tryptophan metabolites in bEECs. (**A**) The level of *IGFBP1* mRNA after bEECs were treated with kynurenine. (**B**) The mRNA levels of *AHR* and *CYP1B1* after treatment with kynurenine. (**C**) The mRNA levels of *AHR* and *CYP1B1* after treatment with Tryptophan. *P < 0.05. Every experiment was repeated at least thrice independently. Con, control; Kyn, L-kynurenine; Trp, tryptophan

As a critical tryptophan metabolite, kynurenine is AHR’s endogenous ligand and the most robust activator in the kynurenine pathway [21]. BEECs were treated with kynurenine. *IGFBP1* expression was significantly upregulated by kynurenine (Fig. 3A). Kynurenine could promote *AHR* expression (Fig. 3B). Meanwhile, kynurenine treatment also upregulated *CYP1B1* of the AHR target gene (Fig. 3B). Furthermore, tryptophan could stimulate the expression of *AHR* and *CYP1B1* (Fig. 3C).

### Effect of tryptophan/kynurenine on the AHR pathway in bEECs

**Fig. 4:**
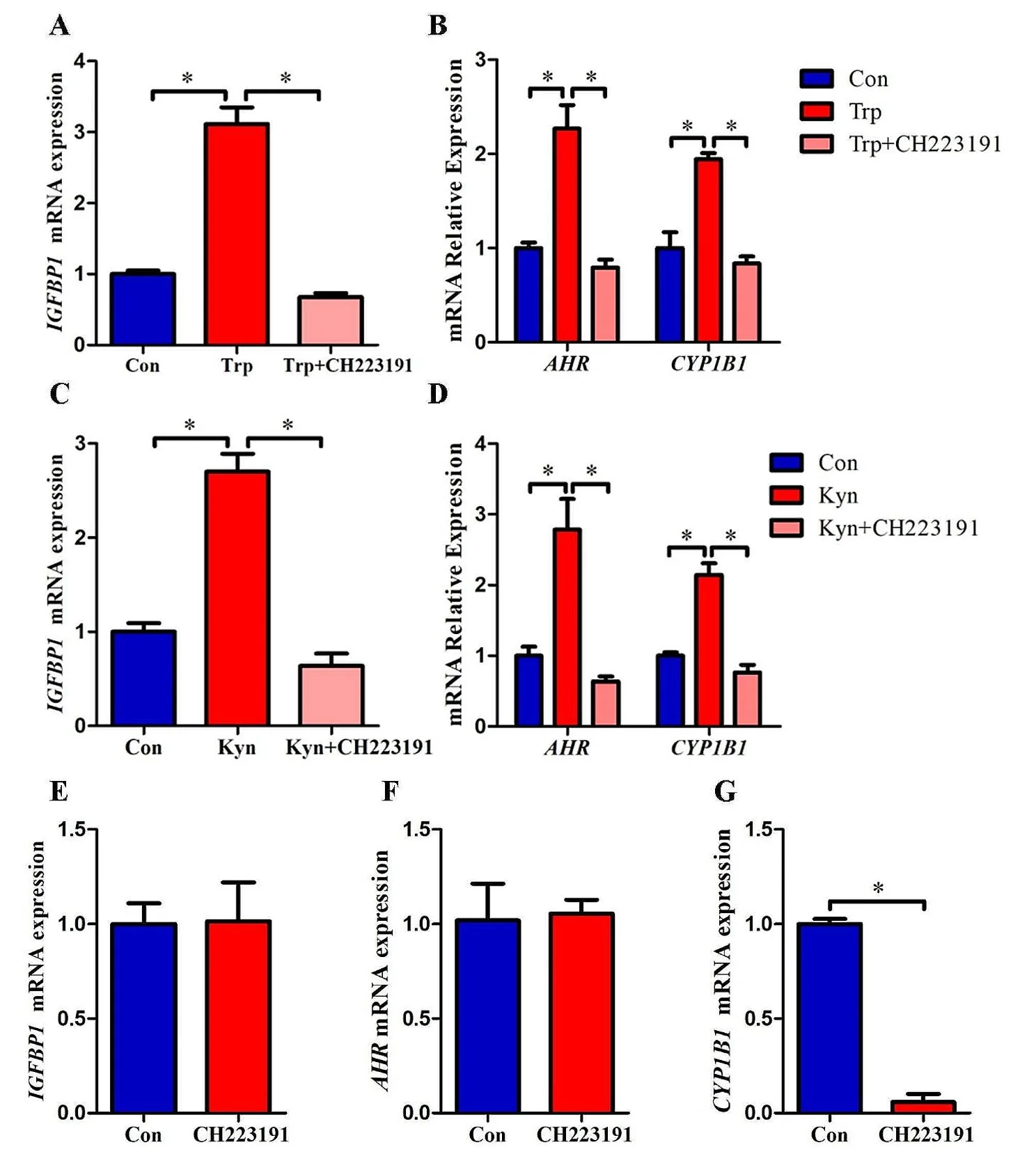
Effects of tryptophan and kynurenine on AHR and its target genes in bEECs. (**A**) The level of *IGFBP1* mRNA after treatment with tryptophan and CH223191. (**B**) The mRNA levels of *AHR* and *CYP1B1* after treatment with tryptophan and CH223191. (**C**) The level of *IGFBP1* mRNA after treatment with kynurenine and CH223191. (**D**) The mRNA levels of *AHR* and *CYP1B1* after treatment with kynurenine and CH223191. (**E-G**) *IGFBP1*, *AHR*, and *CYP1B1* mRNA levels after treatment with CH223191. *P < 0.05. Every experiment was repeated at least thrice independently. Con, control; Kyn, kynurenine; Trp, tryptophan; CH223191, AHR inhibitor

We investigated the role of the AHR in tryptophan-induced IGFBP1 by suppressing AHR with the specific AHR inhibitor CH223191. Treatment with the AHR inhibitor CH223191 significantly reduced the effect of tryptophan on *IGFBP1* expression (Fig. 4A). CH223191 treatment also reduced the mRNA levels of *AHR* and *CYP1B1* (Fig. 4B). Similarly, CH223191 abolished the effect of kynurenine on the expression of *IGFBP1*, *AHR*, and *CYP1B1*(Fig. 4C and D). CH223191 did not affect the expression of *IGFBP1* and *AHR* alone, while it decreased the expression of *CYP1B1* (Fig. 4E–G). We also checked the expression of *TPH1*, *IDO1*, *TDO2*, *SLC7A5*, *AHR*, and *CYP1B1* in the bovine uteri of healthy and repeat breeders. All these genes were decreased in the repeat breeder compared with healthy bovine (Supplementary material 2). Taken together, these findings indicated that tryptophan and kynurenine regulated *IGFBP1* expression by activating the AHR pathway.

### PGE2 regulated the expression of IGFBP1 through the AHR pathway

**Fig. 5:**
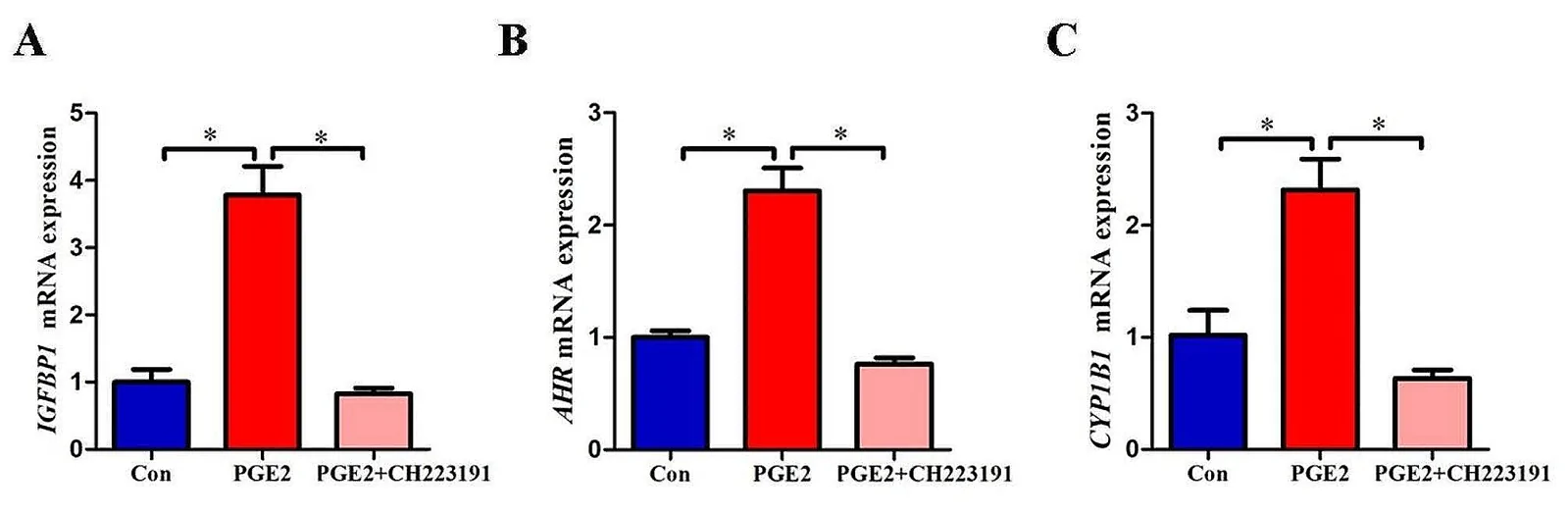
Effects of PGE2 on AHR pathway in bEECs. (**A**) Effects of CH223191 on PGE2-induced *IGFBP1*. (**B**) Effects of CH223191 on PGE2-induced *AHR* expression. (**C**) Effects of CH223191 on PGE2-induced *CYP1B1* expression. *P < 0.05. Every experiment was repeated at least thrice independently. Con, control; CH223191, AHR inhibitor

COX2 plays a vital regulatory role during implantation and development of the conceptus in ovine and bovine [23–25]. As a biomarker for embryonic implantation, PGE2 may be involved in regulating endometrial receptivity, blastocyst expansion, and developmental competence during the window of implantation [25, 26]. To determine whether PGE2 alters uterus receptivity, we measured the level of *IGFBP1* in bEECs. PGE2 stimulated *IGFBP1* expression (Fig. 5A). However, PGE2-induced *IGFBP1* was inhibited by CH223191 (Fig. 5A). PGE2 treatment also increased the expression of *AHR* and *CYP1B1*, which was remarkably suppressed by CH223191 (Fig. 5B, C). These data suggested that PGE2-induced *IGFBP1* promotes uterus receptivity through activating the AHR pathway.

### The regulatory relationship between PGE2 and tryptophan-kynurenine pathway

**Fig. 6:**
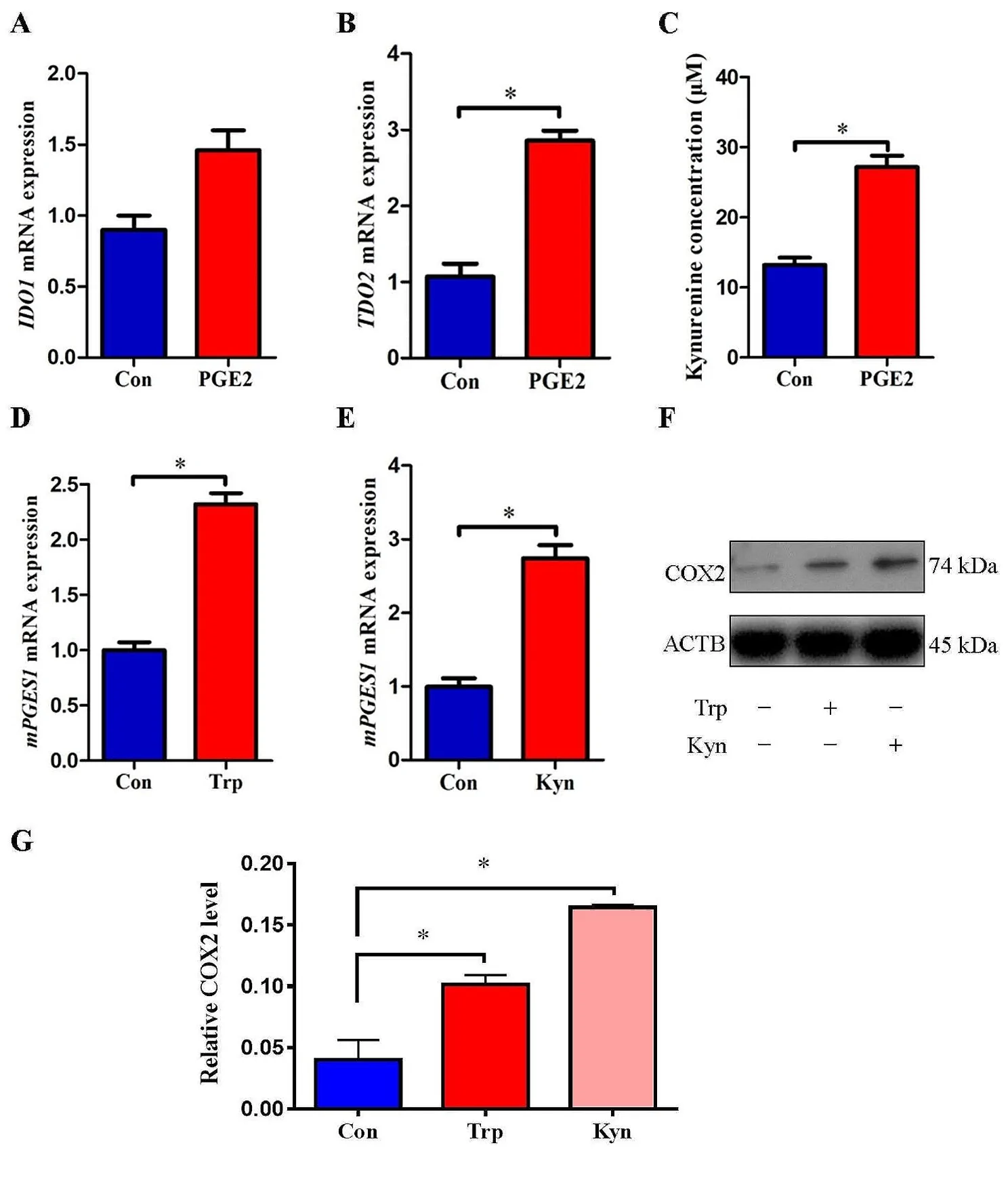
The regulation between PGE2 and tryptophan-kynurenine pathway. (**A**) The level of *IDO1* mRNA after treatment with PGE2. (**B**) The level of *TDO2* mRNA after treatment with PGE2. (**C**) Kynurenine concentration after treatment with PGE2. (**D**) The level of *mPGES1* mRNA after treatment with tryptophan. (**E**) The level of *mPGES1* mRNA after treatment with kynurenine. (**F**, **G**) The COX2 protein level after treatment with tryptophan and kynurenine. *P < 0.05. Every experiment was repeated at least thrice independently. Con, control; Kyn, kynurenine; Trp, tryptophan

We next investigated whether a relationship existed between PGE2 and the tryptophan-kynurenine pathway, which regulated each other. *IDO1* was slightly upregulated, but *TDO2* mRNA level was significantly increased by PGE2 (Fig. 6A, B). Meanwhile, kynurenine concentration was increased dramatically by PGE2 (Fig. 6C). Tryptophan treatment upregulated the expression of COX2 and microsomal prostaglandin E synthase (*mPGES1*) (Fig. 6D, F, and G). Similarly, kynurenine treatment also increased the expression of COX2 and *mPGES1* (Fig. 6E–G). These data suggested that PGE2 might promote tryptophan metabolism toward kynurenine production through increasing expression *TDO2* and *IDO1*, which kynurenine may return increased COX2 and *mPGES1* to promote PGE2 secretion.

## Discussion

IGFBP1 stimulates endometrial receptivity to implantation of the conceptus [8]. The *IGFBP1* mRNA was detected in the luminal epithelium and expressed higher during bovine early pregnancy [6]. The IGFBP expression was lesser for repeat breeder cows with subclinical endometritis than normal cows [27]. Similarly, our study showed that *IGFBP1* expression is higher in normal cow uteri compared with repeat breeders. As a uterine receptivity marker, IGFBP1 low expression may be the reason for repeat breeder cow syndrome. Furthermore, PGE2 promotes the growth and elongation of the ruminant conceptus in a paracrine manner [10]. In this study, PGE2 enhanced *IGFBP1* expression to promote endometrial receptivity. Thus, embryo implantation and fertility are improved and maintained during bovine early pregnancy.

Repeat breeder cows repeatedly fail to be pregnant at least three or more times despite the absence of apparent anatomical abnormalities, normal estrous cycles, or infectious diseases [28]. Nutritional deficiencies, especially Trp deficiencies, can affect the reproductive process in repeat breeder cows [28, 29]. Tryptophan increases the demand for maternal protein synthesis and fetal requirements during pregnancy [15]. Furthermore, the tryptophan content of uterine luminal fluid is stable and increased through the neutral amino acid transporter SLC7A5 during the peri-implantation period of normal pregnancy in cattle [22]. Meanwhile, higher SLC7A5 expression is mounting up transfer tryptophan in the endometrium of the estrous cycle and early pregnancy [22]. Although tryptophan and its metabolites are involved in the maintenance of pregnancy [30], the underlying role is still unclear. This study showed that uterus receptivity may be enhanced by tryptophan and kynurenine via the AHR pathway. Abnormal tryptophan metabolism may affect maternal-fetal interface’s immune tolerance in pregnancy [18]. The lower levels of IDO expression and kynurenine in repeat breeder cows may result in tryptophan catabolism disorder and subfertility [29]. Blocking the IDO enzyme that catabolizes tryptophan along the kynurenine may reduce trophoblast cell proliferation and migration [31]. AHR deletion may be insufficient to support the hormone synthesis needed during pregnancy and lactation, resulting in reproductive deficiencies in AHR ^−/−^ mice [32]. Moreover, AHR was activated by kynurenine and participated in human decidualization during early pregnancy [33]. Therefore, the tryptophan/kynurenine-AHR pathway plays a role in the capacity of the uterus to support pregnancy maintenance.

During the early pregnancy of the mouse, AHR expression was detected in implanted blastocysts and surrounding luminal epithelia and decidual cells [34]. AHR localization was distinct in the endometrium of pregnant compared with non-pregnant rabbits. In pregnant uteri, AHR was found in the whole cytoplasm and nuclei of endometrial epithelial cells, while AHR was only localized in a small cytoplasmic area in non-pregnant rabbits [35]. These may indicate the functional role of AHR in embryonic implantation and uterus receptivity. In porcine, Trp mediates the proliferation of trophectoderm cells by activating the AHR signaling pathway [36]. Kynurenine encourages the NK cytotoxic cells by AHR signal during early pregnancy [37]. Furthermore, Trp and kynurenine activate AHR signaling to stimulate decidualization during human early pregnancy [16]. In our study, Trp and kynurenine also stimulated the AHR pathway to promote *IGFBP1* in bovine endometrial epithelial cells.

PGE2 is involved in every aspect of mammalian female fertility, including ovulation, fertilization, blastocyst implantation, and development [38]. PGE2 was mediated decidualization through PTGER2-dependent PKA activation in vitro human endometrial fibroblasts [39, 40]. Furthermore, PGE2 is involved in maternal recognition of pregnancy in the pig [41]. PGE2 stimulates progesterone-induced IGFBP1 to promote trophectoderm cell migration and attachment in sheep uterus during early pregnancy [42]. PGE2 upregulates TDO-mediated kynurenine release in human malignant glioma [43]. In our study, PGE2 also stimulated *TDO2* to promote *IGFBP1* expression and increase uterine receptivity in bovine.

## Conclusion

In conclusion, our data indicate that tryptophan regulates the expression of *IGFBP1* in bovine endometrial epithelial cells via the PGE2-TDO2-AHR pathway. Meanwhile, PGE2 upregulated kynurenine secretion, stimulating *IGFBP1* expression to increase uterus receptivity in bovine.

## Methods

### Bovine endometrial tissue collection

The Institutional Animal Care and Use Committee of Shanxi Agricultural University reviewed and approved the animal study (SXAU–EAW–2022B.ZT.012005154). Holstein cows housed at a large commercial dairy farm in Jinzhong, Shanxi in China, were used. All animal experiments are conducted in compliance with the ARRIVE guidelines. The present study used healthy cows (4–10 years old, average 600 kg) and repeat breeder cows (4–8 years old, average 620 kg). All animals were fed a total mixed ratio, including hay, silage, and a multivitamin integrator three times a day. The growing environment and feeding management conditions are the same. All were subjected to at least three artificial inseminations. The healthy cows were pregnant and delivered previously. Repeat breeder cows did not become pregnant after three or more breeding attempts, although they have normal estrous cycles. Endometrial tissues from this experiment (healthy (*n* = 10) and repeat breeder cows (*n* = 12)) were collected at the local abattoir.

### Culture and treatment of bovine endometrial epithelial cells

Bovine endometrial epithelial cell line (bEEC, ATCC^®^ CRL-2398™) was obtained from ATCC (Manassas, VA). Frozen bEEC cells were resuscitated onto 100 mm plates and cultured with DMEM-F12 containing 10% FBS and 10% horse serum at 37℃ with 5% CO_2_. bEEC cells were used in experiments in passages 3–7.

Cells (5*10^5^ / plate) were treated with reagents (tryptophan, 500µM; kynurenine, 500µM; PGE2, 20 µM) in 12-well plates for 48 h. For the AHR inhibition study, We preincubated bEEC cells with CH223191 (5µM) for 1 h before the treatment.

### Real-time PCR

Real-time PCR was conducted to analyze the differential expression of genes as previously described [44]. Tissues and cells were extracted to isolate total RNAs with Trizol reagent (TaKaRa, China). The RNAs (62.5 ng/µL) were reversed transcribed into cDNAs with the NovoScript^®^Plus All-in-one 1st Strand cDNA Synthesis SuperMix (Novoprotein, China). ChamQ SYBR qPCR Master Mix (Vazyme Biotech, China) was used for Real-time PCR on the CFX96 Touch Real-Time PCR Detection System (Bio-Rad, USA). The primer sequences in this study are listed in Table 1. The gene expression data of Real-Time PCR were employed to analyze relative changes using the − 2 ^ΔΔCT^ method and normalized to *GAPDH* [45]. Statistical analysis was conducted and followed by the MIQE guidelines [46].

**Table 1:** Primers used in this study

Gene	Primer sequences (5’-3’)	Accession number	Size(bp)
*IGFBP1*	AGCAGCAGAAGGCAGGAGACAA	NM_174554.3	126
	AGACACACCAACAGAGCCCAGG		
*IDO1*	CGCAGCCCAAAGCAGCATCT	NM_001101866.2	134
	TGTGGTGAGCTGGTGGCATGTA		
*TDO2*	GATCACCCGAATGCACCGAGTG	NM_001046313.1	85
	GTCCAAGGCTGTCATGGTCTCC		
*TPH1*	CCAACCCATGCTTGCAGAGAGT	XM_005216077.4	150
	GTAACCAGCCACAGGACGGATG		
*SLC7A5*	TGGAGTGTGGCATCGGCTTCA	NM_174613.2	125
	CTGGCACAGGACCGTCGTAGAA		
*AHR*	TAAGCCTGCCTTCGGGTGTTCA	NM_001206026.1	144
	TGCTGCTGCTGCTCCACTGT		
*GAPDH*	ACGGCACAGTCAAGGCAGAGA	NM_001034034.2	120
	CCACCACATACTCAGCACCAGC		
*CYP1B1*	GACTTGACAGGCAGCGTGATGG	NM_001192294.2	111
	GCACTGGTGAGCAAGGATGGAG		
*mPGES1*	CCAAGTGAGGCTGCGGAAGAAG	NM_174443.2	98
	AGCGTTCCACATCTGGGTCGTT		

### Western blot

Western blot was performed as previously described [47]. Briefly, treated cells were lysed in the RIPA lysis buffer (150 mM NaCl, 50 mM Tris-HCl, pH 7.5, 2 mM Na_3_VO_4_, 0.25% sodium deoxycholate, 1 mM NaF, 1% Triton X-100 and complete protease inhibitor cocktail [Roche]). Protein lysates (10 µg) were electrophoresed using 10% SDS-PAGE gel and transferred onto PVDF membranes. Then, membranes were blocked with 5% non-fat milk and incubated with rabbit anti-COX2 primary (1:2000; Abcam, ab179800, Cambridge, MA, USA) antibody or rabbit anti-ACTB primary antibody (1:2000; Abcam, ab8226, Cambridge, MA, USA) overnight. After the membranes were incubated with HRP-conjugated secondary antibody (1:5000, Invitrogen), we detected the signals by ECL kit.

### Kynurenine assay

Kynurenine was measured as previously described [16]. The supernatant of tryptophan-treated cultures was incubated with 30% trichloroacetic acid for 30 min at 50 °C. Then, the supernatant was mixed with an equal volume of freshly Ehrlich reagent (2% p-dimethylaminobenzaldehyde in glacial acetic acid) and incubated for 15 min. The absorbance was measured at 492 nm and normalized with a calibration curve obtained with L-kynurenine.

### Statistical analysis

All results of the experiments were repeated at least three times independently, except for in vivo study. All analyses were performed using the GraphPad Prism^®^ 5.0 software (GraphPad Software Inc., San Diego, CA). The data were presented as the mean ± standard deviation. Statistical comparisons between the two groups were conducted using Student’s t-test. For multiple comparisons, a one-way ANOVA followed by Tukey post hoc test was performed. Significance was set at *P* < 0.05.

## Electronic supplementary material

Below is the link to the electronic supplementary material.

Supplementary Material 1

## Data Availability

All data generated or analyzed during this study are included in this published article.

## Abbreviations

Trp: L-tryptophan
IGFBP1: Insulin growth factor binding protein 1
TDO2: tryptophan 2, 3-dioxygenase 2
AHR: Aryl hydrocarbon receptor
IGFBPs: Insulin growth factor binding proteins
PGE2: Prostaglandin E2
5-HT: Serotonin
TPH1: Tryptophan hydroxylase 1
IDO: Indoleamine 2,3-dioxygenase
TDO: Tryptophan 2,3-dioxygenase
bEEC: Bovine endometrial epithelial cell line
