# Supplementary figures and images for: Tryptophan regulates the expression of IGFBP1 in bovine endometrial epithelial cells in vitro via the TDO2-AHR pathway
Source: BMC Vet Res. 2024 Sep 4;20:390. doi: 10.1186/s12917-024-04191-9 (PMC11373120; doi:10.1186/s12917-024-04191-9)

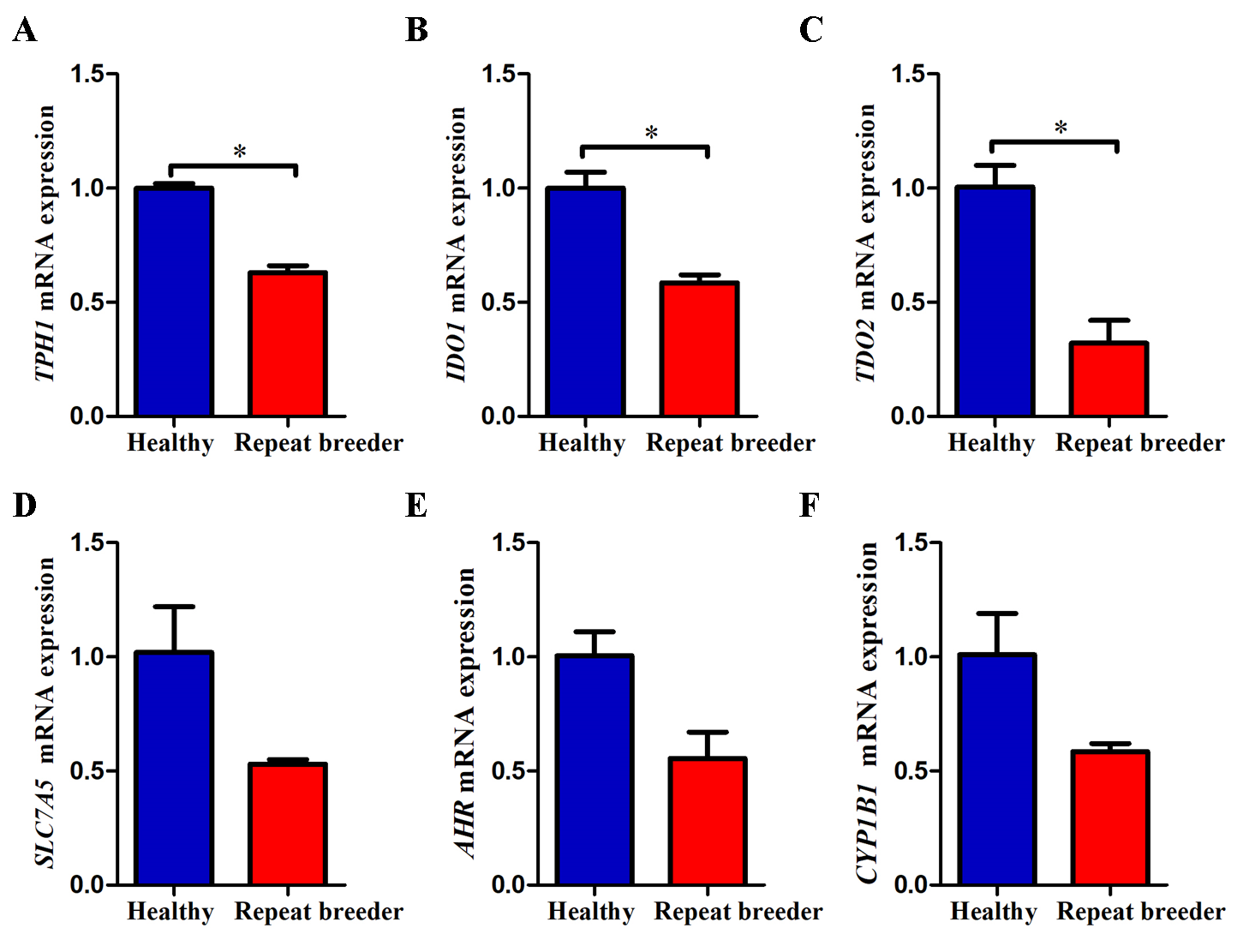

Supplement: Supplementary file 1 — Supplementary Material 1 [file 12917_2024_4191_MOESM1_ESM.tif]
